# Book Reviews

**Published:** 1831-03

**Authors:** 


### CRITICAL ANALYSES.

Quæ laudanda forent, et quæ culpanda, vicissim
Illa, prius, cretâ; mox hæc, carbone, *notamus*.—PERSIUS.

*A Treatise on Pathological Anatomy.* By G. ANDRAL, Professor to the Faculty of Medicine of Paris, &c. *Translated from the French,* by RICHARD TOWNSEND, A.B. M.D. M.R.I.A. and WILLIAM WEST, A.M. M.D. M.R.I.A. Vol. II. —8vo. pp. 830. Dublin: Hodges and Smith. London: Longman and Co. 1831.

IT cannot be necessary that we should premise our notice of this admirable work by many preliminary remarks, in proof of the very strong claims which the author of it possesses to our deliberate attention. The ability and indefatigable industry of M. ANDRAL are as well known as they are highly appreciated. The perfect capability of the translators, also, for the performance of the task they have now completed, has been proved by the fidelity with which they gave to the English reader the first volume of the work. Drs. TOWNSEND and WEST deserve, indeed, much more commendation than is included under the cold term of faithful translators. The language of a foreign author may be clearly understood, and correctly rendered into another tongue; but that which was highly interesting and attractive in the original will become dull and heavy, if the translator does not combine, with a faithful transference of ideas, an agreeable and fluent style in his own language. These gentlemen possess this talent in an eminent degree.

The arduous pathological labours of the French have done much to improve this very important branch of medical knowledge. The numerous and valuable facts which they have contributed, have, however, hitherto been, in a great measure, scattered through so many periodical publications, that they have been comparatively unavailing to the interests of science. To collect these scattered facts, to add to them the results of his own observations, and to arrange the whole into a system of pathological anatomy, form the principal objects of the present work. For this arduous task, the translators truly state, in their preface, few persons could be better qualified than Professor Andral: "placed by acclamation at the head of the pathologists of the French school, he may be considered as the chosen organ of that body, and consequently as expressing the present state of the science in that country." Dr. Townsend, who has himself made, within the last few years, a very considerable number of dissections, assures us that nothing can exceed the accuracy of M. Andral's descriptions; and, as an instance of the extensive scale on which his pathological investigations have been conducted, it is observed, that he introduces his description of the morbid alterations of the lymphatic vessels, by stating that he examined the thoracic duct in *six hundred* bodies. Another circumstance also very materially enhances the value of the work: M. Andral does not confine himself to the irksome task of merely enumerating the various physical alterations that take place in our organs; he endeavours likewise to investigate the origin of these alterations, to explain the mechanism of their formation, and to trace their mutual relation and order of succession. He combats the doctrine, justly termed "absurd" by the translators, that every alteration of the living structure depends on an exaltation of its vital powers. This is a dogma which requires to be opposed in the present day; for there are many practitioners who infer the existence of "increased action" from every pain or ailment they detect in their patients, as well as from every alteration of the natural structure which they find in the dead body. Be it remembered, too, that M. Andral is neither an exclusive solidist or humoralist: "he admits the influence of the solids in producing the phenomena of disease, but he likewise accords considerable importance to the alterations of the fluids." The work, in fact, in the words of Dr. Townsend, "is written in the purest spirit of eclecticism." What is valuable in every theory is selected; but M. Andral is not the slavish follower of any particular sect, or the defender of any abstract and exclusive doctrine.

Having thus briefly, but we believe sufficiently, shewn the spirit and chief purposes of the work, we may proceed to the detailed notice of its contents.

The object of this second part is to describe the morbid states of the several apparatuses, and in particular to consider the lesions they may present with regard to the pathology of the interior.

*Digestive Apparatus;* and first of the "*Diseases of the Portion of the Alimentary Canal below the Diaphragm.*" The first chapter contains an account of the natural appearances of the alimentary canal, concerning which, says M. Andral, there has been hitherto so little agreement, that it is indispensable to determine accurately what is the anatomical condition of the alimentary canal in the healthy state. There are certain circumstances, under the influence of which the alimentary canal, though free from disease, may yet present various degrees of red coloration in the dead body. Some of these circumstances may have operated a certain period before death, others only during the last moments, and others soon, or some time, after the cessation of life. Of the causes which operate before death, some are physiological, others pathological.

"Thus, it is an undoubted fact, that, during the process of chymification, the internal surface of the stomach acquires a considerable degree of redness; as well as that the small intestine does the same while the separation of the chyle is taking place in it: any one may convince himself of the truth of these assertions, by examining living animals. But, besides, it has been ascertained, by observation, that this redness, that is produced by digestion, continues after death; so that, on opening the body of any individual that has died while chymification or chylification was going on within him, we shall find those portions of the alimentary canal, in which the process had been taking place, of an unusually high colour.

"The pathological causes are all such as act by presenting some obstacle to the free return of the venous blood from the gastro-intestinal parietes to the right cavities of the heart. There happens then to the mucous membrane of the alimentary canal what happens in the skin of persons who die of asphyxia: in such cases we observe the cutaneous surface, long before death, acquiring a constantly increasing colour, from the venous blood: now, what takes place in the skin must also take place in the intestine. We may assure ourselves directly of this, by examining a coil of intestine in an animal who is slowly suffocating, when we shall find that, as the respiration becomes more difficult, the coil assumes a more intense and uniform red hue. Lastly, if, as Boerhaave did long ago, we prevent, by a ligature, the circulation of the blood in the trunk of the vena portæ, we shall observe the whole of the internal surface of the intestines assuming a fine red tinge, which is compared by Morgagni to the colour of cochineal; and sometimes even blood transudes through the parietes of the distended vessels, and fills the intestine. These facts being known, it is only drawing the conclusion from them to establish that, every time the blood cannot return freely from the capillaries of the intestinal mucous membrane to the venous trunks, that membrane will continue coloured after death. Hence arise the various shades it presents in cases of strangulated hernias, for instance, or of obstructions of the liver, of tumors situated on the course of the principal divisions of the vena portæ, of the obliteration of the vein itself by old coagula, and, lastly, of organic affections of the heart. If, however, there was but little blood in the body, whether through defect of sanguification, or in consequence of recent copious bleedings, a considerable obstacle to the venous circulation would produce a less intense coloration of the alimentary canal, than that which would arise from a slighter obstacle existing in a person whose vessels contained a great deal of blood a short time before death." (P. 9.)

The red tinge of the gastro-intestinal parietes, in consequence of some mechanical obstacle to the venous circulation, presents various degrees of intensity.

The pathological sciolist will do well to consult this chapter: he will be taught by it "that the gastro-intestinal mucous membrane may be indifferently white or red, without either of these colours necessarily indicating that the membrane had been in a morbid state." It is important to know, also, that, after life has ceased, new causes arise, which tend to produce new modifications in the colour of the intestines, and to inject some parts of it much more strongly than they were at the moment of death.

"The causes of redness produced after death may be reduced to two principal ones: one, the weight of the blood; and the other, its transudation through the parietes of its vessels." The first of these causes begins to act immediately after death, and some ingenious experiments of MM. Trousseau and Rigot are adduced to show the reality of its existence. As soon as a certain space of time has elapsed after death, a new cause of coloration begins to act: putrefaction begins, and the blood contained in the vessels, both large and small, of the gastro-intestinal parietes exudes through the membranes of these vessels, and is effused, in variable quantities, into the surrounding tissues, especially into the submucous cellular tissue.

"On this extravasation of the blood depend, for instance, the red spots almost always observed in the stomach, along the veins of its great extremity, when the body is opened more than six and thirty or forty hours after death. These spots, thus assembled along the course of the vessels, are sometimes isolated, and sometimes grouped together, and running into one another; and in this manner mark the surface of the stomach with streaks and bands of various figures. If, after having observed the stomach in this condition, we leave it, and examine it again at a later period, we find that the redness has increased, and that, moreover, it appears in a new form: it no longer exists solely along the vessels, but the whole surface of the stomach presents a tinge, which has a constantly increasing tendency to become uniform; and a period at last arrives when all the membranes, having become soaked with blood, are equally red: they may then have a tint almost similar to that which we observe on the internal surface of the arteries when stained by the contained blood. This kind of redness, formed after death, cannot, however, proceed to such a high degree, unless in cases where a certain quantity of blood existed in the vessels of the stomach at the moment of death; and as, from the effects of gravitation, that fluid accumulates towards the great extremity of the stomach in particular, it follows that it is there we should see the redness from transudation most strongly marked." (P. 19.)

The period at which this transudation commences depends, of course, upon the commencement of putrefaction, which is variable, and governed by many circumstances. The spleen, also, may suffer some blood to transude through its parietes, and this may then soak into the adjacent portion of the stomach, and impart its colour to it.

Of the colours produced in the mucous membrane of the stomach and bowels, by the causes enumerated by the author, "some cannot be in any way confounded with that resulting from inflammation; others differ from it only by characters which are often but feebly marked." The colour of this membrane presents, also, some different shades, according to the part examined, and the age of the subject inspected. The natural thickness and consistence of the mucous membrane of the alimentary canal are as important to be determined as its colour. In its natural state, it is far from being equally thick throughout. M. Billard has proved that the *maximum* of thickness exists in the duodenum, and the *minimum* in the colon." This membrane may be modified in its consistence, as well as in its colour, after death. This kind of softening has been principally observed long after death, when there were already signs of putrefaction in the body, and in a very short period after death. In the first cases, the membrane loses its consistence but slowly. After enumerating a few, and but a few, of the facts which have been advanced by various English physiologists, to shew that the parietes of the stomach may be softened and perforated by the gastric juice after death, M. Andral concludes by expressing his opinion, that they are neither sufficiently numerous, nor circumstantially enough detailed, to confirm the conclusion drawn from them. As both the isolated and aggregated follicles that are distributed over the gastro-intestinal mucous membrane play a principal part in its diseases, it is of importance to determine accurately what varieties of appearance they may present, without yet ceasing to be in their physiological state. Upon this subject some interesting comments are made.

*Of the Alimentary Canal in a state of Disease.* The preliminary observations to this chapter so clearly shew the honest and pure philosophic spirit with which M. Andral conducts his work, that we cannot refrain from extracting them. In the modern system of book-making, "to hint a doubt," is a rare example of real wisdom. When the actual state of our knowledge will not admit of positive assertion, we too frequently find that even practical authors assume the privilege of poetry, and draw upon their fancy, rather than admit that they have something yet to learn.

"In many instances we shall find that some of the alterations, which belong to the morbid condition, so exactly resemble some of the appearances of the healthy state, as modified by one of the causes already enumerated, that it will be impossible for us to distinguish them. We must then be content to remain in doubt, until new facts give us more light. At other times, we shall meet with conditions of the alimentary canal which we shall not hesitate to recognize as the results of disease; but frequently another question will then arise, as to the nature of these unequivocally morbid states. Do they result from irritation, or, in other words, from an increased organic action of the tissue in which they are situated? Or do they, on the contrary, depend on a diminution of this same organic ation? Or do they arise from neither of these causes, but merely from a perversion of the nutrition? We shall sometimes be able to resolve these different questions; but sometimes, also, for want of a sufficient number of facts to judge from, we shall leave them undecided. For, nothing tends more to give a false direction to a science than presuming to anticipate facts, and attempting to solve a problem of which we do not yet know all the elements." (P. 41.)

M. Andral objects to designate, by the common title of *acute or chronic gastro-enteritis,* the various lesions he describes in this chapter; as, in his opinion, by so doing, he would have prejudged the solution of more than one question, that still remains undecided. Most truly does he state, that this expression has lost its value, from its having become too general. He describes separately the different lesions which have latterly been comprehended under this generic term, and we shall find, he says, that they are always lesions of circulation, nutrition, or secretion.

LESIONS OF CIRCULATION. 1. *Hyperæmia* of the Alimentary Canal.*

"Hyperæmia of the alimentary canal is generally confined to the mucous membrane. Nothing is more common than to find, underneath a portion of this membrane of an intense red colour, the subjacent cellular tissue perfectly white, and the other coats equally devoid of colour. Hence it follows that we cannot judge of the state of redness or paleness of the intestinal canal from examining it only externally: it frequently happens that, in such cases, its parietes appear pale, and the different tunics external to the mucous membrane are really so; but the membrane itself may be of a more or less intense red, without its being perceived until the intestine be opened." (P. 45.)

Gastro-intestinal hyperæmia assumes a variety of forms, but the following species are particularly pointed out.

"In the first species, the light can still be seen through the membranes, between the vessels, which are disposed in a form more or less finely arborescent.

"In the second, the light is totally intercepted and the opacity complete. In these two species the redness that appears on the internal surface of the intestine is disposed in dots, patches, spots, streaks, or bands. It is sometimes lost insensibly, and sometimes ceases abruptly.

"Again, hyperæmia may be divided into three kinds, according as it is situated in the capillaries particularly, in both capillaries and larger vessels, or, lastly, in the larger vessels only.

"Of these three kinds, the first belongs almost exclusively to a state of irritation, and is an almost certain proof of its existence. The second belongs equally to a state of irritation, and to a state of congestion from a mechanical cause that has acted during life or after death. The third kind but rarely depends on this last cause; but yet neither does it announce a state of irritation similar to that which produces a hyperæmia of the capillaries solely. In fact, when the congestion is thus confined to some of the tolerably large vessels that are distributed in or under the gastro-intestinal mucous membrane, we have reason to think it belongs to a state of irritation that is on the decline; and we may even admit that in such cases all irritative process has completely disappeared, and that the blood which is still found accumulated in some vessels, appears there only because these vessels, having been distended by the unusual quantity of blood that traversed them as long as the irritation con-

* M. Andral objects to the term "inflammation," and does not employ it. "Inflammation," he says, in his preface, "being a complicated morbid condition, it is preferable, in my opinion, to describe separately each of the lesions, the combination of which constitutes the 'inflammation' of authors." "Hyperémie" is, therefore, a term used by M. A. merely to denote an increased quantity of blood in the capillary vessels: its classic derivation is obvious.—EDITOR.

tinued, remain passively dilated after all irritation has disappeared. This is what often takes place in the mucous membrane of the eye, in which, long after the capillaries of the conjunctiva have ceased to admit blood, some large red vessels still continue to appear on that membrane. It then frequently happens, that it is by applying substances of a more or less stimulating nature to the conjuctiva that these vessels are forced to resume their natural dimensions, and to get rid of the blood which constantly tends to dilate them. May not this help to account for the success which sometimes attends a tonic mode of treatment, when employed towards the end of certain kinds of irritation of the alimentary canal?" (P. 48.)

*Anæmia of the alimentary canal,* although not a rare lesion, has hitherto, it is said, attracted but little attention. Its anatomical character is an extreme paleness of the canal, either throughout its whole extent, or in some parts only. It is usually accompanied by a more or less considerable attenuation of the gastric or intestinal parietes. It is seldom observed but in the bodies of persons who have died exhausted by various chronic diseases, or who sink while convalescent from a severe fever. In this last case, it sometimes happens that, at the same time that we find the alimentary canal completely devoid of blood, we discover in it ulcers which are as pale as the surrounding tissue. M. Andral has also met with this coexistence of anæmia and ulceration in several young children, who, after having suffered from diarrhœa, had gradually wasted away, and died comatose.

LESIONS OF NUTRITION. *Hypertrophy of the Alimentary Canal.*

"This hypertrophy may exist simultaneously in the different coats of the stomach, or be confined to some of them, or even may involve but one of the anatomical elements of the mucous membrane, such as the follicles. In these different cases, the gastro-intestinal parietes assume very varied aspects, so different, indeed, that they have often been considered varieties of diseases. Frequently, also, the real nature of the alterations produced by hypertrophy of one or more of the coats of the stomach or intestines has been mistaken; for instance, some have considered as new tissues, developed in the substance of the gastro-intestinal parietes, these parietes themselves, though having undergone no change but simple hypertrophy in some of their coats. Thus, the name of cancer has been given to a mere thickening of the submucous cellular tissue, &c.; as well might the name have been given to the thickening of the cellular tissue which surrounds old cutaneous ulcers, or to the induration which affects the interlobular cellular tissue in certain diseases of the lungs." (P. 56.)

Real hypertrophy of the mucous membrane must be distinguished from the thickening it may present in the case of simple hyperæmia, in which more blood than usual stagnates in the mucous membrane, which becomes tumefied, but its nutrition has not been really increased.

"When the gastro-intestinal mucous membrane is really in a state of hypertrophy, it is at the same time harder and more resisting than in the natural condition: it may be removed in large shreds without tearing, and the cellular tissue which separates it from the muscular coat remains uninjured beneath.

"Hypertrophy of the mucous membrane, both of the stomach and intestines, may be accompanied by different shades of colouring, which it is important to notice. Thus, it may exist, 1, with retention of the usual colour of the membrane, which is a rare case; 2, with different degrees of redness; 3, with a slate-coloured tint; and 4, with a brown or even black tint." (P. 57.)

When hypertrophy of the mucous membrane occupies only isolated points, it produces, on the internal surface of the stomach, or intestines, elevations, projections, patches, and tumors, of various forms, sizes, and textures. The varieties of appearance of partial hypertrophies depend on the differences they may present in form or texture.

"The mucous membrane, while shooting up above its surface, or vegetating, may either preserve its usual consistence, become much harder, or, lastly, present several degrees of softening: it may be pale, or display all possible shades of colour; it may receive but little blood, or be traversed by numbers of vessels, and by veins which, from their gorged and dilated state, sometimes resemble varices, or, from their interlacement and the facility with which the blood escapes from their interior, represent a sort of erectile tissue. According to these different textures, different names have been given to the circumscribed hypertrophies of the gastro-intestinal mucous membrane; they have been denominated vegetations, fungi, polypi, scirrhi, &c." (P. 59.)

In the alimentary canal of children, the follicles are naturally more apparent than in the adult. In adults, whenever the internal surface of the canal is found studded with very apparent follicles, we are to consider their developement as a morbid state.

"The follicles of the intestinal tube may be much more apparent than ordinary, and project above the free surface of the mucous membrane, without being in a true state of hypertrophy. This is what happens in those cases where, in consequence of an acute or chronic inflammation of the canal, the follicles spread over it become the seat of a more or less considerable active hyperæmia. Being then gorged with blood, they become tumid, and form on the internal surface of the intestine a greater or less number of small conical elevations, which frequently exhibit a central orifice that has been often taken for an ulcer. These are isolated where the follicles are so; and are confluent, and form by their assemblage large patches elevated above the level of the mucous membrane, where the follicles are aggregated; constituting the *dothinenteritis* of M. Bretonneau." (P. 56.)

After the cessation of the hyperæmia, the follicles increase in bulk, or else retain the bulk they had acquired while congested; and they are then in a real state of hypertrophy. The orifice of the enlarged follicles often retains its natural size; in other cases, it grows wider; and lastly, in others, it grows narrower, tends to become obliterated, and even actually does become so. The enlargement of the orifice is sometimes so considerable, that the mouth of the follicle becomes as wide as the bottom; in which case, the follicle might easily be mistaken for an ulcer. In other cases, while the parietes of the follicle become enlarged and thickened, its orifice diminishes, and becomes effaced. The result is, that the fluid secreted in its interior constantly accumulates, and voluminous tumors are thus gradually formed; such as are often found in the intestines of horses. M. Andral has seen some in the stomach of these animals as large as an orange. Their real nature might easily be mistaken, but that, in most cases, the orifice of the follicle may be discovered by attentive examination, and a probe introduced, so as afterwards mechanically to enlarge it.

"We must take care not to confound with follicles in a state of hypertrophy certain small whitish bodies which are sometimes found scattered on the internal surface of the intestines, and which at first sight greatly resemble such follicles. These are nothing but rudiments of valvulæ conniventes, on or between which they are situated. Some tufts of the villi, of a deader white than those around them, might also be sometimes mistaken for follicles." (P. 69.)

In the next section, hypertrophy of the tissues subjacent to the mucous membrane of the alimentary canal, is described. This kind of hypertrophy may affect, 1, the cellular tissue interposed between the different coats of the gastro-intestinal parietes, and, 2, the muscular coat.

"Writers have long described under the name of scirrhus of the stomach or intestines a certain state of those parts consisting merely of an increase of thickness and density in the cellular membrane which, in the natural condition, separates the mucous membrane from the muscular tunic. Any one may convince himself of this by following the hypertrophy of this kind of cellular tissue through all its stages. In the first place, in many cases of chronic diarrhœa, where the mucous membrane of the great intestine has undergone various kinds of alterations, the cellular tissue which lines it is often found much more apparent than usual; in such cases, it is sometimes several lines in thickness, so as of itself to surpass that of all the other coats taken together: it is hard, of a pearly white, and without any blood-vessels; sometimes there may be observed in it fibres or plates more or less regularly arranged; and sometimes it exhibits nothing but a homogeneous texture, pretty like that of imperfect cartilage. As long as this cellular layer is of little thickness, it is considered only as indurated cellular tissue, similar to that which surrounds old cutaneous ulcers, or appears between the pulmonary lobules in certain cases of chronic affections of the respiratory organs. There is often interposed between the mucous and muscular coats, from the commencement of the colon to the rectum, a white layer of from half a line to two lines in thickness. This, again, is considered merely as an induration of the submucous cellular tissue.

"But, when the hypertrophy is more considerable, and at the same time more circumscribed; when, in short, it forms in some one point of the intestinal tube a real tumor which raises up the mucous membrane, it is then no longer denominated induration, nor hypertrophy, but scirrhus. In this case, however, the alteration is the same as in the preceding ones; the cellular tunic has become ten times as thick as natural in a circumscribed point, instead of becoming twice as thick throughout a great extent: there lies the whole difference. The same disposition, texture, and anatomical elements, are to be found in the whitish layer that lines the mucous membrane of the great intestine, as in the bulky tumor that obstructs the cardia or the pylorus. Writers, then, may, if they please, give the name of scirrhus to such a tumor, provided that they come to an understanding as to the meaning of the word, and acknowledge that in this case the scirrhus is not a new tissue, without any thing analogous in the healthy state, and created, like an entozoon, in the parietes of the stomach or intestines; but that it is simply the result of a hypertrophy of the submucous cellular tissue, which may either continue to be the sole lesion, or may subsequently become complicated with other alterations of nutrition or secretion. In fact, a new structure often does develope itself in the cellular tissue thus affected with hypertrophy; numerous vessels make their appearance in it; it becomes divided into lobes, or hollowed into cells or areolæ; and, lastly, various morbid secretions take place." (P. 69.)

M. Andral has often carefully examined the nerves of animal or organic life that are distributed over the gastro-intestinal parietes, but he has never been able to discover the least alteration in them.

"However, M. René Prus has had an opportunity of observing a case in which one of the branches of the pneumogastric was evidently affected with hypertrophy, near its termination on the stomach. The subject was a man of fifty-two years of age, who fell a victim to a tedious disease of the stomach, during which he exhibited all the symptoms of a so called cancerous affection of that viscus. The mucous membrane was found but little altered; the submucous cellular tissue was of a dead white, of the consistence of lard, and considerably thickened; and the muscular coat was very much affected with hypertrophy. These different alterations existed in the body of the stomach, for an extent of about three inches transversely: the pylorus and cardia remained free. A tumor, which had been discovered during life, was the result. In this terminated the right œsophagean branch of the pneumogastric, which, from the cardia to the superior part of the tumor, had increased to twice its bulk, without change of colour or consistence; it then entered the tumor, where it was no longer possible to follow it." (P. 88.)

*Atrophy of the alimentary canal* may take place either in the whole of the layers that compose the gastro-intestinal parietes, or in one only.

*Softening of the alimentary canal* is one of the most frequent alterations observed in the parietes of one or more of the coats which form them. The mucous coat most frequently becomes soft: it may be reduced to a liquid pulp, the consistence of the other coats not being diminished. Sometimes all the membranes are simultaneously affected with softening, and then the parietes of the stomach, or of some part of the intestines, may be very easily torn. Softening of the digestive mucous membrane is particularly remarkable in the stomach, and this affection may prevail through the entire extent of the stomach; "and there are cases in which it has been well ascertained, that the mucous membrane of almost the whole of that viscus, having been reduced to a diffluent pulp, had disappeared. From the cardia to the pylorus, the internal surface of the stomach, almost throughout, presented nothing but the cellular layer, which was thus immediately in contact with the ingesta." (P. 93.) Softening of the mucous membrane of the stomach is accompanied by various shades of colouring; for the particular description of which we refer to the work.

"Softening of the mucous membrane of the stomach is a lesion that may be produced either by an acute or by a chronic process. The introduction of irritating poisons into the stomach often causes it to take place in a few hours, and the mucous membrane becomes at the same time strongly injected.

"In many chronic diseases, especially of the lungs, a period arrives when the patients lose their appetite, and digestion becomes difficult; as for the rest, they have neither nausea, vomiting, nor pain in the epigastrium, nor is the appearance of the tongue altered. The lesion most frequently found in the stomach under such circumstances is a softening of the mucous membrane, together with one of the shades of colouring above described.

"Sometimes, instead of commencing slowly, and announcing its presence only by slightly marked symptoms, the softening takes place by a more acute process, and its existence is indicated by pain in the epigastrium, vomiting, and redness of the tongue. In such cases it is found to be accompanied by a bright redness of the membrane.

"Softening of the mucous membrane of the stomach appears to me to be a common affection in old people whose digestion becomes disordered, their health having previously been very good. Their appetite first diminishes, they then lose it entirely, and, soon after, they begin to feel the greatest dislike to all kinds of food. They experience a constant feeling of uneasiness and weight, rather than actual pain, in the region of the stomach; and their tongue, which is usually natural, or else more or less thickly coated, grows red and dry occasionally. This state may continue for several months; the pulse then becomes more frequent, a considerable emaciation takes place, the strength rapidly declines, and the patients die without shewing symptoms of a serious affection of any organ up to the last moments. On opening the body, there is nothing found but a more or less considerable softening of the mucous membrane of the stomach, with or without injection of its tissue.

"We have just seen that in many cases where, after death, the gastric mucous membrane is found softened in various degrees, there has not been any pain in the epigastrium during life; or, at most, the patients have complained only of a kind of disturbance, uneasiness or weight in that region. Some, however, have for a long time had very severe pain in the epigastrium, and yet no other lesion could be discovered in their stomachs, after death, than in those of the others. Here, then, is another case where the difference of the symptoms is in nowise explained by the difference of the alterations.

"What has been said of the softening of the mucous membrane of the stomach applies exactly to the softening of that membrane in the rest of the intestinal canal. I shall therefore content myself with drawing the reader's attention to those cases of pretty frequent occurrence in which the eye cannot detect any perceptible lesion on the internal surface of the intestines, the cæcum and colon especially, although there has been a copious diarrhœa during life. Here, one would at first be inclined to allow that the affection took place without any lesion of the intestine; but, on applying the scalpel to the mucous membrane, and attempting to detach it, he will in most cases be struck with its extreme softness, and he will sometimes even find it reduced to a pulp devoid of all traces of organization. Thus, then, in certain chronic diarrhœas, the only alteration observed in the alimentary canal is a white softening of the mucous membrane of the end of the small, or of the great intestine." (P. 96.)

*Softening of all the layers of the gastro-intestinal parietes.* In some cases we find that the mucous, muscular, and serous coats, together with the various interposed cellular layers, have lost their usual appearance, and become soft, friable, and in a manner dissolved; and they not unfrequently resemble a kind of transparent jelly, which scarcely preserves the appearance of an organized texture. M. Cruveilhier has described this remarkable lesion with the greatest accuracy, and has given to it the name of "*gelatiniform softening.*" In such cases, before touching the parietes of the stomach or intestines, we might take them to be quite sound, as their consistence alone is modified. This general softening of all the coats may, like that of the mucous membrane only, take place, 1, their natural colour being preserved; 2, they being remarkably pale; 3, their tissue being red.

"The splenic portion of the stomach is the part where the species of softening at present under consideration has most frequently been observed. I saw it involving the whole of the stomach in two children, of whom one had taken some sulphuret of potash a few months before his death, and the other had died with the various symptoms which characterise acute hydrocephalus. In the first of these cases, the whole of the parietes of the stomach resembled the pulpy part of a red cherry; there had been constant vomiting during life. In the second, the child was in good health, when, without any known cause, he was seized with vomiting, which, after having continued for two days, was succeeded by convulsions, and at last by a comatose state, in which he died, five or six days after the first attack. The stomach was found to be softened to such a degree through its whole extent, that wherever pressure was made on its parietes, they were reduced to a pap, and torn; but this stomach, very unlike the other, was remarkably pale throughout; and yet the progress of the disease had been acute." (P. 100.)

The following very interesting questions are discussed at some length: viz. whether softening of the parietes of the stomach or intestines, when confined to a single coat, or extended to all, takes place only during life? whether there are not cases where it is merely produced after death? and whether, in the first place, it may not be sometimes the result of putrefaction? It is an undoubted fact, that, in proportion as a body putrifies, the tissues composing it gradually lose their consistence, as is particularly evident in the various parenchymatous organs, but the mucous membrane does not begin to soften until signs of advanced putrefaction have appeared in other parts of the body; whence, M. Andral thinks, we should conclude, that, when the mucous membrane is discovered to be ever so little softened, without there being as yet any traces of putrefaction in the body, it is not to be attributed to that cause.

"It may, then, be established as a general principle, that any softening observed in the parietes of the alimentary canal on opening the body at the usual period after death, should not be considered as the result of putrefaction. But, are we to conclude from this, that every time it is met with, it is to be considered as having taken place during life? To this question, some reply that a considerable softening of the parietes of the stomach may take place after death, not from putrefaction, but from other causes which do not act until life has terminated. In a thesis maintained in Germany, by Camerer, before Professor Autenrieth, (Stuttgard, 1818,) we find an account of several examinations of bodies of rabbits and cats, that had been killed while in very good health; in which it is stated that in all of them the great extremity of the stomach was softened, and its parietes were in a manner dissolved, and even, when a sufficient time had elapsed after death, perforated. Yet, when these examinations were made, the bodies did not shew the least sign of putrefaction; while, on the contrary, in the body of a dog already putrefied, there was no trace of softening in the stomach. We see, then, that Camerer discovered this affection in the bodies of healthy animals, before decomposition had commenced; but did not find it in animals that had died of disease, nor in those that died a lingering death. It happened only in those cases where an animal in good health was killed in a short time after the reception of food into the stomach, and during the process of chymification. Can this be, as Hunter taught, owing to the gastric juice, which, having been secreted before death, and collected in the stomach, may, once that life has ceased, dissolve the parietes of the stomach, just as, during life, it dissolved only the organized, though not living substances, contained in that viscus? M. Camerer mentions on this head a very remarkable experiment, which, if it does not prove the action of the gastric juice on the parietes of the stomach, proves at least that certain fluids contained in softened stomachs may produce softening in other stomachs when introduced into them. A fluid collected in the stomachs of two children, that had died of a gelatiniform softening of that viscus, was introduced, in the dose of a drachm, into the stomach of a man not long dead, which was then kept for twelve hours in a moist heat of 20° R. (77° F.) At the end of that period, the membranes of the stomach were found dissolved through to the peritoneum, wherever the fluid had been in contact with them. The same fluid was introduced into the stomach of a living rabbit, without producing any bad effect; and on the animal's being killed, its stomach was found in the most healthy condition. On the contrary, another portion of the same fluid having been deposited in the stomach of the rabbit, after death, its parietes exhibited a pultaceous softening after a certain period. Now if, during life, the nerves distributed to the stomach from the pneumo-gastric and trisplanchnic be cut on each side, and the same fluid be then introduced into the stomach of the animals thus treated, their stomach softens; but this effect does not ensue if the nerves be cut without applying the fluid.

"Several of these facts appear to me to prove clearly that softening of the stomach should be reckoned as one of the alterations which may take place after death in certain cases. There may have been in the stomach during life a morbid affection of such a nature, as to discover itself to us after death by the softening of the stomach, as it did before that period, by certain well-marked functional derangements; since it may have produced such a state of the stomach as to render it capable of softening after death under the influence of causes, which would otherwise have been either not called into action, or else inadequate to produce that effect." (P. 102.)

M. Andral, with his accustomed candour, confesses that the cause which produces softening of the alimentary canal during life, is far from being evident in every case. We must acknowledge that this condition appears to be often connected with a process of irritation; but that the existence of such a process is not sufficient to produce it, and that, consequently, it is neither the sole nor the necessary cause of it.

*Ulceration of the alimentary canal* is next considered. In every part of it, ulceration may result, from acute, as well as from chronic irritation.

"In this respect, however, there is a remarkable difference to be observed between the stomach and the intestines, properly so called. In the stomach, ulceration is very rarely produced by acute irritation; it scarcely ever occurs there but after a chronic irritation of long continuance. In both the small and the great intestine, it is frequently produced by chronic irritation, as in the stomach; but, besides, it very often results from acute." (P. 112.)

The peculiarities which intestinal ulcerations present in their number, size, form, and direction, are pointed out.

"Are ulcers of the alimentary canal susceptible of cicatrization? The best way to answer this question is by giving an account of what has been observed in persons that died during convalescence, and in whose intestines were found ulcers in different states, from that in which they were still in full action, to that in which they were beginning to disappear.

"In a man who died on the fifty-third day of a severe disease, during the course of which there had been a copious diarrhœa which continued to the last moment, the other morbid symptoms also becoming chronic, I found on the internal surface of the ileum and cæcum several ulcers, with elevated margins, and brown, wrinkled bottoms. But, in a few, the bottoms were on a level with the margins, which were pale; in some of these, the bottom consisted of the ordinary submucous cellular tissue; in others, of an extremely delicate web, continuous with the margin, and situated above the cellular layer. This web seemed to be the commencement of the reproduction of the mucous membrane.

"A woman was attacked with fever, with a brown dry tongue, diarrhœa, and delirium; from which she recovered. When fully convalescent, in consequence of a surfeit, the fever and diarrhœa returned, great prostration of strength succeeded, and death ensued. On dissection, there were found, towards the end of the small intestine, 1, some greyish patches, of a somewhat oval form, and slightly elevated above the level of the internal surface of the intestine; (these were Peyer's glands in a state of tumefaction;) and, 2, in other parts where these glands usually exist, some spots, which were likewise nearly oval, but were evidently below the level of the surface, and consisted of a very soft, delicate, rose-coloured membrane, which when put in water appeared to be without villi, and resembled a portion of the bronchial mucous membrane. Some other scattered points were discovered to be also without villi, while just beside them they were very apparent.

"It seems, then, that in the two cases just mentioned, we have instances of the reproduction of the mucous membrane, which, under such circumstances, at first makes its appearance without villi, like than of an excretory duct.

"In another case, in a person who died a few months after having had a continued fever with copious diarrhœa, I found, about a foot above the ileo-cæcal valve, a remarkable puckering of the mucous membrane, which was at the same time of a brown colour: the exact resemblance between this and certain cutaneous cicatrices, induces me to think that it also may have been the result of cicatrization. In the same person, Peyer's glands were very conspicuous, and the mucous membrane had lost its villi in some circumscribed spots.

"I have on three or four occasions found a puckering resembling that just described, in the stomach of persons about whose previous diseases I was unfortunately unable to procure any information. In one of these, a slip of mucous membrane, about four or five lines in length, passed over the puckering, and adhered by its extremities to the surrounding portion of the membrane." (P. 120.)

Dr. Craigie* observes, in speaking of ulceration of the different coats of the stomach, that "the most important point to be known is, that these ulcers may cicatrize." In proof of the fact, he refers particularly to the post-mortem appearances detected in the body of the late M. Beclard.

Under the title of "*Perforations of the alimentary canal,*" the student in pathology will find much valuable information. This subject is replete with interest, inasmuch as a question has very frequently arisen in courts of justice, as to the probable cause of these perforations, when there was reason to suspect that poison had been given, although positive proof of the fact was wanting. We are anxious to direct the especial attention of practitioners to this subject, as we know instances, in this and other countries, where the testimony of medical

* Elements of General and Pathological Anatomy, p. 685.

men has placed the lives of innocent persons in jeopardy, in consequence of their having very erroneously maintained that such appearances, particularly when they were detected, as they often are, in persons who had died suddenly, and in the plenitude of health, could only arise from the agency of some poisonous substance.* In many cases, as M. Andral states, the causes which produce the perforation of the gastro-intestinal parietes are manifest, and their manner of acting is easily explained. "But what explanation are we to offer, when the stomach and intestines become suddenly perforated, in the midst of a very good state of health? What are those perforations that are denominated by authors *spontaneous?* The term was adopted at a time when it was thought that every perforation of the gastro-intestinal parietes was the result of poisoning with corrosive substances.

"Some cases were observed in which perforations had occurred without the intervention of any external cause to account for them, and they were consequently termed spontaneous. If we read over again the observations that were published on this kind of perforation, we shall find that they may be ranged in two classes. Some of them relate to cases in which, before the occurrence of the perforation, there had been more or less strongly marked signs of an acute or chronic affection of some part of the alimentary canal; while, on opening the body, the perforation was found in or near membranes that were ulcerated, softened, or, in short, altered in some way or other. In others, there is nothing of the kind: individuals in perfect health are suddenly attacked with very severe pain in the abdomen; they die in a few hours; and, on opening the body, an effusion is found in the peritoneum, and a perforation in some part of the parietes of the stomach, without any other lesion of that viscus, except, in a few instances, a pulpy softening around the perforation. Are we to say with Hunter and many modern pathologists, that such a perforation may be owing to the corrosive quality the gastric juice can sometimes acquire, by virtue of which it in a manner dissolves the parietes of the stomach? This hypothesis has already been discussed. Shall we, then, say that it is the result of a process of irritation that has become suddenly developed in some part of the canal? But, this is just as much an hypothesis as the supposition of Hunter.

"We must acknowledge, then, that there are some perforations, the cause of which is far from being yet perfectly known to us. Be it what it may, an important question presents itself: are there any certain anatomical characters, by means of which a so-called spontaneous perforation may be distinguished from one which is owing to the action of a poison? and, are such characters to be found

* Vide Dr. EBERMAIER's paper;—in our Journal for October 1828, p. 302, and November 1828, p. 422.—EDITOR.

in the form of the perforation itself? I think not; for I have seen the perforation affect the same varieties of form (sometimes the circular, with a smooth margin, and sometimes the irregular, with a fringed or torn margin, presenting shreds of various membranes,) both in men in whom it was not produced by poison, and in animals in which it was. Shall we then draw these distinctive characters from the appearance of the parts surrounding the perforation? They are not at all more certain; for, whether there has been poison in the case or not, the surrounding parts are alike red, disorganized, softened, and gangrened. Lastly, traces of violent irritation may be found in the rest of the stomach, in both cases equally. When, however, there are numbers of sloughs in several spots in the stomach, there is strong ground to suspect the presence of poison, because such sloughs are but very seldom observed, in any other case. When, on the contrary, there is no other lesion observed in the stomach but the perforation itself, the probability is that there has not been any poisoning; for it is not easy to conceive how a corrosive substance introduced into the stomach, could act but upon one point precisely. The thing would not, however, be absolutely impossible. It follows from this discussion, that anatomical examination, while it often furnishes no useful information to enable us to distinguish a so-called spontaneous perforation, from a perforation from poison, sometimes affords a greater or less degree of probability, but never perfect certainty." (P. 128.)

Perforations in the intestinal tube may occur in every age. They have been observed in children, scarcely a few days or weeks old. It is evident that perforations of the gastro-intestinal parietes may establish various kinds of communication between the exterior and interior of the stomach or intestines.

Changes of capacity in the alimentary canal may arise from various causes. M. Andral affirms, from his own experience, that the assertion that, in *colica pictonum*, the intestines are so strongly contracted, as to present a much smaller cavity than natural, is by no means founded on fact. The muscular coat of the intestine may be contracted in different manners. A portion of intestine, quitting its natural situation, becomes inverted, and enters the adjacent portion.

"One of the most remarkable phenomena presented by introsusceptions, is the complete separation of the introsuscepted portion, without any escape taking place into the peritoneum; the detached portion passes out by the anus, and a perfect cure follows. Hevinus saw two cases of this description; one of the patients passed twenty-three inches of the colon, and the other, twenty-eight of small intestine. The preparations were presented to the Académie de Chirurgie. M. Cruveilhier shewed the Societé de la Faculté de la Médecine a coil of intestine eighteen inches long, with some of the mesentery adhering to it. It was passed by a man who, for several days, had had all the symptoms of strangulated hernia, which ceased as soon as it came away. I had an opportunity of examining a preparation, sent to the Académie Royale de Médicine, by MM. Bouniol and Rigal; and ascertained it to be a portion of the small intestine, about thirty inches in length, with a small bit of the mesentery adhering to it. The subject of the case had had an excessive surfeit, after which, he was seized with all the symptoms that announce strangulation of the intestine: he had besides, in the right iliac region, an uneven tumor, which was very sensible to the touch. At the end of twelve days, he passed the portion of intestine and mesentery in question; upon which, the symptoms disappeared, and he recovered, retaining only a feeling of pain in the right iliac region. In three months after, having eaten an enormous quantity of cherries, he was attacked with the symptoms of peritonitis, and died. It is a pity the body was not examined: there would, probably, have been found a rupture of the cicatrice, which must have been formed where the expelled portion of intestine had been detached. In other cases, however, there has been an opportunity of ascertaining the condition of the alimentary canal in persons who died, in a longer or shorter time after having expelled a portion of intestine by the rectum. A case of this kind is related by Hevinus: a man, who had all the symptoms that characterize internal strangulation, passed a long portion of intestine, composed of the whole of the cæcum, six inches of the ileum, and six of the colon: twelve days after this, he died. On opening the body, the following appearances were observed: The cæcum was wanting; the ileum opened into the colon, with which it was intimately united: at their point of junction was a narrow aperture, which led to a collection of pus situated in front of the right kidney, and circumscribed on all sides by false membranes. Another fact of this kind is recorded in the second volume of the Bulletins de la Societé Philomatique. It relates to a man who died forty-four days after having passed, by stool, a portion of the small intestine, sixteen inches in length. M. Dumeril saw the preparation. On examination after death, the following peculiarities were discovered: 'The two extremities of the intestine were perfectly united, and appeared as if their edges had been bevelled, and then fitted exactly to each other. They had contracted strong adhesions with the peritoneum at their junction; nevertheless, their cavity was not sensibly diminished, even at the cicatrice. The deficient portion belonged to the jejunum and ileum.'" (P. 146.)

Introsusception of the intestines has been observed at every age. The parietes of the intestine may, by thickening, produce obliteration of its cavity in some particular point: the same effect may be produced by a vegetation growing on the internal surface of the mucous membrane. A foreign body, a calculous concretion, an accumulation of fecal matter, or a bundle of worms, in the intestinal cavity, has often proved, says M. Andral, a complete obstacle to the course of the contents, and produced all the symptoms of strangulation. Obstacles may exist without the strangulated portion of intestine.

"These obstacles may be owing to an irregular disposition, either of the peritoneum, or of the intestinal tube itself.

"The causes of strangulation proceeding from the peritoneum are the following:

"I. Perforation of the omentum.

"II. Perforation of the mesentery.

"III. Division of the omentum into several strips, which adhere to the abdominal parietes, and are liable to entangle some of the coils of intestine.

"IV. Accidental fræna, extended like arches:

"*a.* From one portion of intestine to another;

"*b.* From a portion of intestine to another organ: thus, M. Esquirol saw a strangulation produced by a frænum extending from the ligamenta lata of the uterus to the rectum. (It was a preparation presented to the Académie Royale de Médecine.)

"*c.* From a portion of intestine to the abdominal parietes;

"*d.* From the omentum to another part of the abdomen;

"*e.* From one of the abdominal viscera to another.

"V. The mesentery being rolled up like a cord, and involving coils of intestine.

"The causes of strangulation proceeding from the intestinal tube itself, are the following:

"I. The compression of one portion of intestine by another. One of the most remarkable cases of this kind is that mentioned by Dr. Gendrin.* In a child, six months old, who had from his birth suffered under habitual constipation, and continual vomitings, he found a portion of the transverse colon situated between the vertebral column and the duodenum, which confined it like a ligament.

"II. The perforation of a coil of intestine, and the escape of another coil, introsuscepted into the first, through the aperture so formed. In this case, the introsuscepted coil is strangulated by the margin of the perforation.†

"III. The unusual length of the appendiculum cœci, whereby it gets twisted round the ileum, and thus produces strangulation.

"IV. Preternatural adhesions of the free extremity of the appendiculum, by which an arch or ring is formed, which readily produces strangulation, if a portion of intestine gets entangled in it.

"V. The twisting of a diverticulum around a coil of intestine.

"VI. Adhesions of the free extremity of the diverticulum, which, then becoming an arch, may compress any portion of intestine that may happen to slip under it. However, it is not always the entan-

* "Archives de Medecine, vol. viii. p. 494.

† "Bulletin de la Societe Medicale d'Emulation pour 1822. Case by M. Martin Solon.

gled portion of intestine that is strangulated; it has sometimes been known to produce strangulation in another portion, which, by adhering to the free extremity of the diverticulum, completes the ring of which this latter forms a segment.

"The various causes of intestinal strangulation, that have now been enumerated, may occur suddenly or slowly. In the former case, very serious affections, which rapidly prove fatal, make their appearance all at once, without any premonitory symptoms. In the latter, there are observed, for a longer or shorter space of time, for months, or even years, various symptoms, which are all connected with the existence of a greater or less obstacle to the free passage of the contents of the alimentary canal.

"Some of these causes do not necessarily produce strangulation. Thus, all the varieties of fræna that have been described are found in dead bodies, without such an effect having taken place; but then a single contractile movement of a coil of intestine, made in a certain direction, would have been sufficient to cause it during life." (P. 150.)

The next section describes various kinds of congenital malformation, &c. of the alimentary canal below the diaphragm.

Lesions of Secretion frequently occur in the alimentary canal, and form in it various diseases. Morbid secretions on the mucous membrane are of two kinds: the one being the natural secretions, merely increased in quantity; the other, new secretions. The natural secretion of mucus, the perspirable vapour, and the gases, may be excessive in quantity. Sometimes there is a prodigious quantity of mucus on the internal surface of the stomach or intestines. In place of mucus, there is occasionally found in the intestinal tube an enormous quantity of a fluid resembling water. This seems to be merely the vapour usually exhaled by the mucous membrane, which, being increased in quantity, appears in the fluid form.

"The stomach is, in like manner, filled with a great quantity of serous fluid; its size is considerably increased; it occupies the greatest part of the abdominal cavity, and at the same time its parietes are exceedingly attenuated. I saw myself a case of this kind, in which the great curvature of the stomach almost reached the pubis, and the fluid that filled its enormous cavity could be seen through the transparent tissue of its delicate parietes. In this case, the stomach presented no appreciable lesion; very unlike another enlarged stomach already mentioned, in which the pylorus was also diseased." (P. 172.)

In the healthy state, the alimentary canal always contains a certain quantity of gases, the nature of which has been ascertained by chemical analysis. In the morbid state, these gases sometimes increase in quantity to such a degree as to distend the intestine, and produce several bad effects. The colon suffers most frequently in this manner. An increase of the gaseous secretion of the canal often accompanies a state of acute irritation of the mucous membrane. M. Andral, however, has more than once found the intestine greatly inflated, without presenting any lesion after death, in persons who had died of an acute affection of the brain. In one instance, which occurred in our own practice, we found the ileum and colon remarkably distended with gas: the last intestine was as large as an ordinary adult stomach. The only other morbid appearance observed, was a slight partial redness of the mucous coat of the intestine, and this had most probably been produced by the free exhibition of drastic purgatives.

"If any one should doubt that an increase of the gaseous secretion of the alimentary canal could take place, without any lesion of circulation or nutrition in its parietes, I shall bring forward the remarkable fact of tympanitis being so frequently observed in hysterical women? What do we observe in such cases? an indubitable modification of the innervation, and a tympanitis accompanying this modification, sufficiently often to be fairly considered as one of its results. We should then consider what may occur with respect to the secretion of gas, in typhoid fevers, where innervation certainly plays a very great part, whether it be primary or secondary. The mere chemical reaction of the alimentary substances introduced into the digestive passages, is often a cause of tympanitis. Veterinary surgeons are well aware that, by feeding sheep in a certain manner, there is such a developement of gases produced in their paunch, that they die of suffocation, if the gases be not suffered to escape, by plunging a trocar into it through the parietes of the abdomen." (P. 174.)

Blood, in different quantities, is occasionally found in the stomach and intestines. Various causes may give rise to this effusion: among others, certain states of the blood itself, in which it is so changed as to have an universal tendency to escape from its vessels. This occurs in certain cases of poisoning by absorption, and in typhous fevers: in these, the frequent hemorrhages from the digestive mucous membrane no longer result from a primary alteration of the membrane itself, but are one of the elements of a morbid state, which exists wherever the blood has access. The black vomit in the yellow fever, M. Andral conceives to be one of the most striking instances of such hemorrhages. In most cases of intestinal hemorrhage, nothing peculiar is discovered in the parietes of the intestinal canal, except that they are more or less strongly injected with blood. In two cases of hæmatemesis, which were examined by Professor LALLEMAND,* no peculiar lesion was discovered: in each, the mucous membrane of the stomach, for an extent of three or four inches, was puffy, and of a crimson colour, permitting the blood to exude from it on pressure, and so easily torn, that at the least touch it came off in shreds. Nearly the same appearances were observed by ourselves in the bodies of two females, who died, somewhat unexpectedly, with the same disease. Intestinal hemorrhage is not very uncommon in new-born infants: M. BILLARD saw fifteen cases of it in one year. Some of these children were plethoric; others, on the contrary, pale and weakly. "Thus, then, hemorrhages may take place from the surface of the intestines, as it does from every other mucous membrane, without there being necessarily any serious lesion of the tissue from which the blood escapes."

There is another more uncommon kind of gastro-intestinal hemorrhage, in which there is found, in the internal surface of the stomach or intestines, a solution of continuity of a large vessel, which is then seen gaping in the interior of the canal. Such an occurrence, however, M. Andral states, is rare: there are, at most, but five or six well-authenticated instances of it.

*Melanosis.* Instead of exuding blood, the gastro-intestinal mucous membrane sometimes exudes a black matter, in which most of the elements of that fluid are discovered, but which differs from it in appearance, and in the circumstances under which it shews itself. The alimentary canal of certain animals, in the fish tribe particularly, is naturally coloured by this matter. "In that of man, and other mammalia, it is to be considered as a preternatural secretion, but yet as one which, in many cases, no more gives rise to any morbid phenomena, than are produced in the lungs by the black matter which so often stains both the parietes of the air-vessels and the interlobular cellular tissue." M. Andral mentions two cases, in one of which the internal surface of the large intestines, and in the other the stomach, was tinged with a black colour as deep as that of Indian ink. In neither had there been, during life, any well-marked sign of gastric affection. The mucus usually secreted, in variable quantities, in the internal surface of the alimentary canal, may be replaced by various other secretions; for the detailed account of which we must refer to the work.

In article 5, some interesting observations are made upon

* Première Lettre sur l'Encephale, p. 93.

tha state of the alimentary canal, in the different cases where its functions are deranged.

Notwithstanding the length to which this analysis of M. Andral's work has extended, we have as yet gone through but a third part of the volume. It would have been impossible, either with credit to ourselves, or satisfaction to our readers, to compress within a few pages even a sketch of all the very important matter it contains. Upon a future occasion, we shall give an account of the remainder of the work. P.326.

---

*The Dublin Hospital Reports and Communications in Medicine and Surgery.* Vol. V.—8vo. pp. 640. Hodges and Smith; Dublin, 1830.

(Continued from our last Number.)

THE next paper we have to notice is by Dr. EVORY KENNEDY: it is entitled "*Physiological and Practical Observations on the Utero-Placental Circulation, and the Phenomenon of Placental Soufflet, with its utility in detecting the existence of Pregnancy, and the Death of the Fœtus in Utero.*" On applying the ear, either naked or with the stethoscope, to the abdomen of a pregnant woman, provided the pregnancy be at a sufficiently advanced stage, we shall, in almost every instance, observe, at some part of the uterine tumor, a peculiar sound, bearing more or less resemblance to that emitted by the action of bellows, which has, by its discoverer, KERGARADEC,* been termed the "placental soufflet," from the circumstance of his having observed it at that part of the outer wall of the uterus, corresponding to where this body was attached internally. As this appears to Dr. K. an important fact, both in a physiological and pathological point of view, he has devoted much attention to its consideration, and now gives us the result of his observations. He states that, in our examination for this sound, as well as that of the fœtal heart, we may sometimes be able to detect them through the patient's clothes, whilst she stands erect; but, from the attrition of these causing confusion, the best method, perhaps, in every case, and certainly in the early months, will be to examine the patient lying on her back in bed, with a sheet spread over the abdomen.

The comparative merits of mediate and immediate auscultation have excited discussion. M. FODERA,† although he

* Mémoire sur l'Auscultation, appliquée à l'Etude de la Grossesse, &c. &c. Paris, 1822.

† Journal de Physiologie, par M. Magendie, t. ii. p. 116.

recommends the use of the stethoscope in ordinary cases, from motives of delicacy, says, that in general the sounds can be heard more distinctly with the naked ear. Siebold,* in speaking, as he calls it, of the hearing trumpet, says, with this instrument, notwithstanding all the pains he took, and his frequent experiments, he heard nothing. Such was also the case with many other accoucheurs, who made the same attempt. On the contrary, he asserts he heard with the naked ear, as well the double beat, as the fœtal heart's action, and also the whizzing sound of the single or placental pulsation. The result of Dr. Kennedy's observation is, that, although cases may occur, in which (upon the plan long since adopted by Wrisberg, of pressing the cheek to the abdomen, and thus having the advantage of the sense of touch, as also that afforded by the weight of the head fatiguing the abdominal muscles, in addition to the signs of auscultation,) this practice may be admissible; yet he thinks, as well from motives of delicacy and cleanliness, as from the advantages which the stethoscope affords us in exploring parts of the abdomen, which we could not reach with the unassisted ear, that the general use of this instrument is preferable; and he is convinced that, whichever we accustom ourselves to, will afford us the greatest facility in detecting the sounds. The placental soufflet does not always exhibit the same unvarying characters; yet these are sufficiently marked to render it recognisable in almost every case. It assumes all those varieties described by Laennec under the head of bellows sound.

"These sounds are, from the distention of the uterus, and consequent facility of examination, easily discovered in advanced pregnancy: and although not so loud and sonorous in the earlier stages yet to the practised ear are quite as distinct. I have not observed any of the above-mentioned sounds peculiar to particular stages of pregnancy, having detected them indifferently throughout all. This phenomenon is perceptible over an extent of surface greater or less, according to circumstances; it may have its seat in any part of the uterus, and cases will occur, although, if proper precautions be had recourse to, very rarely, where we shall not be able to detect it." (P. 234.)

Dr. Kennedy next endeavours to explain the causes which produce the sound in question, and from which these irregularities proceed, by some very ingenious remarks upon the vascular structure and circulation of the utero-placental system. The placental soufflet he conceives "to depend on the trans-

* Abbildungen aus dem Gesammtgebiete der Theoretisch-praktischen Geburtshülfe, &c., von E. C. J. von Siebold. Berlin, 1829.

mission of blood through the arteries of that part of the uterus to which the placenta is attached, and which we designate the placental portion, deeming it at the same time possible that the passage of the blood through the arterial tubes and cells of the maternal part of the placenta above described, may have some effect in the production of it. Lest this should appear a matter of assumption on my part, I shall enumerate the facts, founded on cases, observations, and experiments, which led to this conclusion." (P. 241.)

The placental sound is present in pregnant women, only when the utero-placental circulation exists, and ceases when the vessels which serve to sustain this function are no longer pervious.

"A fact which we can ascertain by examining a woman shortly before parturition, when we observe this phenomenon in full energy; and again, when the uterus is empty and perfectly contracted after delivery, or when the fœtus having died in utero, an obstruction in this system of vessels is produced, in all which cases not the slightest vestige of the phenomenon can be discovered. The sound is distinctly periodical, alternating with intervals of cessation, and corresponding in every case with the mother's pulse at the wrist, with which it is synchronous. The sound is always heard in that part of the uterus where the placenta is or has been attached; a fact which I have frequently proved by manual examination, when it has become necessary to introduce the hand into the uterus to remove the placenta, as well as by ocular demonstration after death. The circumstance also of this sound existing in the cow, the only animal in which I have as yet detected it, exhibiting similar characters to those already enumerated, goes a considerable way in proving that it depends on the utero-placental structure, which we have already seen in this animal, approaches near to that of man. Add to the above the anatomical structure of this part of the human uterus, as already explained, so peculiar, and unlike any other structure in the body." (P. 242.)

Of the practical benefits to be derived from a knowledge of the phenomena of soufflet, the most important is that of the assistance which it will afford us in the detection of pregnancy, when taken either singly or in conjunction with the fœtal heart's action.

Dr. Kennedy is very far from admitting that auscultation is the only unequivocal evidence of pregnancy: he justly maintains, that we yet stand in need of some aid, to assist us in arriving at accuracy of diagnosis in doubtful cases of pregnancy; and he has no doubt that auscultation will go a considerable way in affording us this, and he even hopes that, in ordinary cases, it may be substituted for vaginal examination.

"When a perfect placental soufflet exists in any part of the abdominal tumor, particularly if the fœtal heart's action also can be detected, we may pronounce the woman pregnant. When it cannot be discovered, and that of the fœtal heart is perceptible, there is reason to conclude that the placenta is attached to the posterior wall of the uterus. This, however, is a rare occurrence; and when we do not succeed in our examination of the anterior part of the abdomen, by causing the patient to alter her position, and exploring the iliac and lumbar regions of either side close to the ilium and back towards the spine, we shall very seldom indeed fail of hearing it. We have already seen that intermissions will occur in this phenomenon, upon what cause depending I cannot say. Kergaradec endeavoured to explain them by the change of position assumed by the fœtus, with regard to the placenta: how this will produce the effect he does not shew, possibly it might do so if the fœtus, in altering its position, so pressed against the placental part of the uterus, as to prevent the transmission of blood through its vessels. In such cases the cessation of this sound is not permanent; therefore, by repeating our examination, we shall succeed in discovering it. I should mention that uterine action suspends it in some altogether, whilst in others it converts it, during the pain, into an abrupt sound or pulsation, and these are almost the only cases in which I have observed this omission to occur.

"Where we can with facility ascertain the action of the fœtal heart, as in the advanced stages of pregnancy, discovering the placental sound, although it is satisfactory as an additional diagnostic, does not become an object of the same importance as in the earlier stages, where the heart's action, being weaker, frequently eludes our observation: it is also of consequence in cases where the uterus contains a dead child, which are often most perplexing in their nature, from the contradictory symptoms that coexist. Pronouncing decidedly in cases of doubtful pregnancy, before the period of quickening, has always been looked upon by practical men as a hazardous undertaking; and although I do not mean to say that we can now, with the assistance of auscultation, be enabled to pronounce with certainty in every case before this period; I with confidence state, that where we detect a placental soufflet, which in the great majority of cases is practicable at a considerably earlier period, taken in conjunction with the other symptoms, it will tend much to strengthen the opinion of the presence of this state. The coexistence of the fœtal pulsation with the soufflet, of course, decides the question; but with regard to the latter, taken separately, I will even go so far as to say, that were all the other symptoms of pregnancy absent, and that this sound could be distinctly perceived, I should at least withhold my opinion until a sufficient time had elapsed to place the matter beyond a doubt. In support of this assertion, I will mention a case which occurred to me about twelve months since.

"I was sent for one morning by a lady, who came over clandestinely from the sister kingdom. The statement she gave me was as follows. She had for some months been in the habit of receiving the attentions of a gentleman for whom she had formed an attachment, but unfortunately fell a victim to her father's caprice, who, after countenancing this attachment, suddenly withdrew his consent to their union, and insisted on her marrying an individual of his selection. Ten weeks had elapsed from the time of her first giving way to illicit intercourse, when she consulted me; during which, two monthly periods had passed without the usual menstrual discharge. About ten days before my seeing her, in consequence of some active exertion, a discharge of blood took place from the vagina, which lasted for a few days. Her father urged her compliance with his wishes, and she dreading to enter the marriage state whilst there was a possibility of her being pregnant, consulted a medical man of eminence, who, after the usual investigation, pronounced that such was not the case: impressed, however, with a painful foreboding of the true nature of her state, although she had no further symptom of pregnancy than that already mentioned, she determined on obtaining further advice, and under pretence of visiting a friend in the country, came over to Dublin. On the most accurate examination, I could ascertain no further grounds for suspicion, than the presence of a remarkably distinct soufflet, which was discoverable on pressing the end of the instrument in the pubic region over the uterus. Relying on this, I gave her to suppose that there was a strong likelihood of her being pregnant, although I could not actually pronounce such to be the case. The result fully justified the confidence I reposed in this as a means of diagnosis; for exactly nine months from the period when she calculated, she gave birth to a child." (P. 257.)

Dr. K. has not succeeded in detecting the placental sound in any case, until after the second month from impregnation; but he has frequently done so in the tenth, eleventh, and twelfth weeks.

"A woman named Devereux, who had been under my care in labour eighteen months before, called to consult me for a slight attack of pneumonia. She mentioned that her menses had not appeared for the last two months. I therefore examined her with the stethoscope, and detected clearly the placental soufflet, although no uterine tumor was observable. Dr. Collins, who also examined her, expressed his astonishment at its distinctness at that early period. I gave this woman reason to suppose it possible that she was pregnant, of which she had not the slightest anticipation. Its accuracy, however, was attested by her coming into hospital on the 7th of March, 1830, in labour, and being delivered of a living child the day following, exactly twenty-nine weeks from the period at which we had examined her.

"To this it may be objected, that this woman went beyond forty weeks, as the birth of the child was the only means I had of calculating the period of impregnation; but I could adduce several cases in which I detected it at an equally early period, in all of which, it is not likely, that the mothers should have carried their children beyond their natural time." (P. 261.)

The following case is interesting, inasmuch as it shews under what perplexing circumstances we may be assisted in our diagnosis by the stethoscope.

"I was requested by my friend Mr. Dwyer, at that time attending the hospital, to see with him a case of dropsy; he said he had some reason to suppose the woman pregnant, but could not quite satisfy himself on the subject. I therefore visited her in company with this gentleman, and Dr. Whitestone. Her abdomen was enormously distended, and fluctuation very evident: the extremities and lower part of her body were œdematous. She stated that she had been attacked with general dropsical symptoms, preceded by extreme debility whilst nursing her last child, about a year and a half previously, but had experienced no local distress. She had been living with her husband during this time; her menstrual discharge had been very irregular, being absent for several months at a time, and again returning, she did not observe it at all since August last; did not quicken, as usual in her former pregnancies, but thought she had lately observed something like the obscure motion of a child. Nothing satisfactory could be ascertained from a vaginal examination, from the extremely swollen state of the parts, neither could the most accurate abdominal examination with the hand enable us to detect any resemblance to an enlarged uterus. The conclusion, therefore, that we arrived at was, that with the ordinary means it would be impossible to pronounce this woman pregnant. Recourse was then had to the stethoscope, when the placental soufflet, full and distinct, was without difficulty observed by all, extending high up on the right side of the abdomen: the fœtal pulsation, although it could not then, was afterwards discovered remarkably small and obscure. At times this could not be heard, from the distance at which it lay from the surface, both the cavity of the abdomen, and, as it afterwards appeared, the uterus, being distended with an immense quantity of fluid. The placental sound, on the contrary, from the parietes of the uterus approaching so much nearer the surface, was quite audible, and continued so throughout. As I had it in contemplation to perform paracentesis, I begged Dr. Collins and Mr. Houston to see her with me, who also satisfied themselves as to the phenomena above stated.

"She was afterwards brought into the Lying-in Hospital, where, in consequence of the extreme difficulty of breathing and exhaustion which set in, it was deemed expedient by Dr. Collins, in consultation with Drs. Labatt and Colles, that premature labour should be induced, which was carried into effect by rupturing the membranes, the os uteri having at this time become accessible, and shewing some inclination to dilate, she was delivered, in twelve hours, of a living child, about the seventh month, which survived its birth forty-eight hours. The mischief which might have arisen from ignorance of the real nature of this or similar cases, is self-evident, and requires no comment." (P. 262.)

Dr. K. cautions those who may be unacquainted with the stethoscope, that they may be deceived by other sounds, either from their resemblance to the placental soufflet, or the effect they have in concealing this.

"Another advantage of importance we derive from our knowledge of the placental sound, is its assisting us in pronouncing on the life or death of the fœtus in utero. The benefits which accrue to us in practice from this are abundantly obvious, and extend not merely to the satisfying our own mind, and that of our patients, but also to the actual preservation of life. Every midwifery practitioner will acknowledge, that, though he is always guided as to the length of time he will attend the issue of a tedious labour, by the state of the mother, and that her safety is his first consideration; yet a period of some, nay many hours, will elapse, in which, were he satisfied of the death of the child, he would not delay a moment in effecting the delivery; and by so doing, I doubt not, the lives of many mothers, whose loss creates a chasm in society, and tends to bring a disgrace on the obstetric art, might be saved, when no detriment could accrue to the already lifeless and innocent cause of its mother's destruction. Of the benefit derivable from auscultation in this respect, I cannot speak in too high terms, and that from having seen it extensively put to the test in the Dublin Lying-in Hospital, where about 2,500 patients are delivered annually, and, of course, frequent opportunities afforded of proving its utility." (P. 267.)

Dr. K. concludes his paper with some other interesting comments and suggestions respecting the use of the stethoscope in obstetric practice.

The next article, by Mr. RYND, *on some of the Affections of the Fingers and Toes, attended with fungous Growths*, is of some practical importance.

*A Case of Ruptured Intestine, with Remarks on some Effects of Contusions of the Abdomen*, next follows, from the pen of Mr. HART.

"July 12th, 1826. An hostler, æt. thirty-six, received a kick from a horse in the abdomen, about eleven o'clock A.M. He was brought to Mercer's Hospital, at six o'clock on the evening of the same day, complaining of excruciating pain over the whole abdomen, with constant vomiting. He had a pale, anxious countenance; shrunk features; pulse 140 per minute, small and feeble; the lower extremities were drawn up; the abdomen flat and rigid, and so tender that he could not endure the slightest pressure over it, even that caused by the weight of the bedclothes.

"He said that, on the receipt of the injury, he fainted, and that, on recovering his consciousness, he felt the pain in his abdomen; the intensity of which had increased every hour. He had eaten no breakfast, but had drunk some porter a short time before the occurrence of the accident.

"Previous to his admission into the hospital, he had been bled, and some purgative had been given him, which was almost immediately thrown off his stomach by vomiting.

"On examination, it was ascertained that he had not made water since he met with the accident, and that he was unable to do so from the pain the effort occasioned. A catheter was passed, and about two pints of high-coloured urine were drawn off, without seeming to have any influence on the abdominal pain.

"Mr. Wm. Daniell, under whose care he was admitted, and who consented to allow me to publish the case, ordered leeches to be applied to the abdomen, their application to be followed by fomentation, and an emollient enema to be injected, and repeated every hour until the bowels were freed. Several stools, tinged with bile, and containing some solid fæces, followed the injection of the second enema: the symptoms, however, suffered no mitigation, and he died, in a state of extreme exhaustion, at five o'clock on the following morning, having survived the accident eighteen hours.

"*Dissection, eight hours after death.* The peritoneum was suffused with numerous small red vessels, and coated over with a thin layer of fawn-coloured lymph, which could be easily scraped off, and in which no red vessels were visible; the intestines and omentum were agglutinated to each other, and to the inner surface of the abdominal parietes in several places, through the medium of this soft lymph; a quantity of serous fluid, tinged with bile, and mixed with the contents of the intestines, was found within the sac of the peritoneum: this was altogether free from any fœtid odour. On examining the intestines, it was found that the effusion had taken place through a rupture in the upper portion of the jejunum, which divided this intestine half across: owing to the retraction of the coats of the intestine, this opening was of an oval form, and surrounded by a raised border, consisting of the everted mucous membrane.* The lymph was in greatest abundance, and the adhesions were most extensive in the vicinity of this opening. There was a large dark-coloured patch on the arch of the colon, produced by an ecchymosis between its coats; but this intestine was unbroken, although distended with fæces, some of which were nearly solid, as if they had been retained there for some length of time.

"Between the layers of the transverse mesocolon, and behind the root of the mesentery, there was a large quantity of dark-coloured coagulated blood, amounting in quantity to about sixteen ounces. This blood had been shed from the smaller branches of the mesenteric vessels, as we ascertained, by a careful examina-

* "As described by Mr. Travers, in his work on Injuries of the Intestines."

tion, that the aorta, and all the large veins, were uninjured." (P. 295.)

The occurrence of rupture of an intestine, without breach of the abdominal parietes, has not, Dr. H. believes, been as yet satisfactorily accounted for. The late Professor Todd conceived that it was owing to the portion of the intestine which suffered, happening to be in a distended state at the time when the contusing force impinged on the surface of the abdomen. Mr. Hart replies to this opinion, by observing, that this particular kind of injury almost invariably happens to the jejunum, where it is continuous with the duodenum, or at some short distance from that point; and, as the jejunum is not usually found more distended than any other portion of the small intestines, it appears obvious that its distention cannot be the reason why it is the part invariably ruptured by external violence.

"Some other cause than simple distention must, therefore, operate in predisposing a certain portion of intestine to suffer the injury in question. It occurs to me, that this may more justly be attributed to the circumstance of the intestine being, to a certain degree, fixed on some resisting surface, at the moment when it receives the impulse of the contusing force. Such is the condition of the upper portion of the jejunum, which at its commencement is fixed to the spine, and which, for some inches lower down, is confined to the vicinity of that part by the shortness of its mesentery. The absence of such contiguous resisting surface, would explain why it was that the arch of the colon, in the case here cited, escaped being torn, although the violence had acted on it with a degree of force sufficient to rupture the blood-vessels between its coats, so as to produce extensive ecchymosis. Every part of the large intestine, however, is not so exempt from rupture produced by external violence, as are the arch of the colon and the more moveable part of the small intestines; the cæcum, for instance, which is usually bound down by the peritoneum to the surface of the right iliac fossa, is occasionally burst by external violence. There is a case of this kind related by Mr. Speer, in the fourth volume of these Reports." (P. 299.)

Mr. H. briefly mentions other serious injuries, which are occasionally produced by contusions of the abdomen.

Dr. Houston contributes some valuable *pathological observations.* The following case, which we extract from his paper, is curious and important.

"*Case of Attempt at Suicide, with Danger of Suffocation by the falling down of the Epiglottis.*

"In March 1828, a servant out of place, residing in Duke street, during a fit of delirium, brought on by intoxication, attempted to destroy himself by cutting his throat with a razor. I saw him in about ten minutes after, and found him almost lifeless. The pulsations of the heart were imperceptible, the pulse at the wrist had ceased to beat, the limbs were cold, and all feeling and consciousness lost. The wound, which was frightfully deep, extended more towards the left than the right ear. The razor had entered between the os hyoides and the thyroid cartilage, and disunited them so completely as to allow the former to ascend with the tongue into the mouth. The pharynx was laid wide open, and the epiglottis severed from its attachments to the tongue and os hyoides, and left hanging by its pedicle to the back of the pomum adami. The carotids had escaped untouched, and the bleeding was inconsiderable. It appeared difficult at the moment to account for the sudden extinction of life; the symptoms were evidently those of suffocation, but the cause was not at first understood. I passed my finger into the wound, and found, to my surprise, that the epiglottis, loosened from its upper and lateral attachments, had fallen back over the rima glottidis, and completely intercepted the passage of air to the lungs. I raised the obstructing body and drew it forwards: the chest soon after began to heave, respiration returned, the heart and pulse again beat, and consciousnes and sensibility were re-established.

"It required some effort of my fingers to hold up the epiglottis, as the air at every inspiration tended to force it back again to its unnatural and dangerous position.

"While thus occupied with the patient, Surgeon Porter entered the room; I explained to him the singular nature of the case, and how between my finger and thumb I held the regulation of the man's life or death. The top of the epiglottis was then brought over the edge of the thyroid cartilage, and secured to its anterior surface by a single stitch. The man in a short time sat up, and attempted to speak, but was unable to articulate. He was taken into the Meath Hospital under the care of Mr. Porter, from whom I learned that he never recovered from the delirium which led to the perpetration of the act, and died in about a week after, of erysipelatous inflammation of the neck and throat.

"The circumstance which had so nearly extinguished the life of this individual the instant after the wound was inflicted, is too plain to require comment; but the fact of its occurrence, and of the facility with which the immediate danger arising from it may be averted, is too important not to be made generally known. A respite of life for even a few hours in cases such as this, may be often of infinite value."* (P. 315.)

*A Case of obstinate and extensive Psoriasis, successfully*

* "Since the report of this case I have observed, in Blandin's Traité d'Anatomie Topographique, the following paragraph, which shews that, if the accident alluded to has escaped notice, the possibility of its occurrence has been at least contemplated. He says, 'L'epiglotte neanmoins, qui est alors coupée, peut par son abaissement sur le larynx causer des graves accidents."

*treated,* is related by Dr. W. West. Practitioners must be well aware of the perplexing and often intractable nature of this disease, and they will consequently be happy to receive any rational suggestions which are likely to render their endeavours to relieve it more successful.

"In June, 1829, Mr. H. W., aged 20, of a robust constitution, was attacked by psoriasis, which first made its appearance on the lower part of the back, then extended to the thighs and legs, and at last covered the scalp, the whole of the body and extremities, with the exception of the face and hands. This eruption did not at first excite much uneasiness in his mind, as he thought it would go off spontaneously; but, when he saw it spreading more and more every day, he applied to me for advice.

"I prescribed Plummer's pill, and the application of an ointment composed of a mixture of tar, citrine, and sulphur ointments, a mode of treatment I had found effectual in other cases of this disease. The eruption, however, still gaining ground, he had recourse to sulphur-vapour and artificial Barege baths, which he persevered in assiduously for nearly three months, with little permanent improvement, although he, at the same time, took a considerable quantity of the liquor arsenicalis internally, in the manner advised by Biett, and had occasionally tried the effects of a lotion of tincture of iodine diluted, and some other reputed specifics.

"At last I consulted Dr. Graves, who advised the antiphlogistic method of treatment recommended by Dr. Duffin, together with a perseverance in the arsenical solution, after the inflammation had been reduced by venesection, aperients, and low diet. We had recourse to these remedies, and continued to apply them for many weeks, but without relief, as the disease, although occasionally mitigated, assumed its former degree of violence, the moment the patient took exercise, or in the slightest degree deviated from the use of vegetable diet: under these circumstances he naturally became impatient, complaining that his strength was much reduced, while little or no progress had been made in curing the disease.

"It was now proposed to omit the use of all remedies for some time, until he should have regained his strength, and his constitution have recovered from the impression made on it by the arsenical solution, &c.; when we proposed to recommence the treatment, but in a different and more energetic manner.

"Dr. Graves observed, that a strict adherence to the antiphlogistic regimen is much more readily enforced when the patient is confined to bed, than when he is allowed to go about; and that the constitution is much less injured by the antiphlogistic treatment, which consequently can be pushed farther, and with more effect; that confinement to bed has also the advantage of guarding the skin from all sources of external irritation, and, by tending to keep it in a perspirable state, accelerates the cure.

"Our patient, a young man of active habits, was, but with difficulty, induced to submit to this confinement. He was placed on a cooling and scanty vegetable diet, was bled several times, and his bowels were kept open by a purgative electuary, consisting of cream of tartar and electuary of scammony. The good effects of this treatment soon became apparent: the redness of the affected parts diminished rapidly, the formation of scales decreased in the same proportion, and in less than five weeks he was almost completely freed from a disease, which had lasted more than six months, and had resisted so many active remedies. He has not since had any appearance of relapse, nor is there any mark or discoloration of the skin where it had been the most severely affected.

"As those affected with psoriasis and lepra are usually persons of strong constitutions, confinement to bed will generally be resisted, as unnecessary and irksome: when, however, the disease is general over the surface of the body, and does not yield to the usual plans of treatment, we may have recourse to it with much advantage." (P. 331.)

There are still some other valuable contributions in this volume, of which we shall give an account in our ensuing number. We are much gratified by the zeal and ability displayed by our professional brethren of the sister kingdom, and we are unwilling to pass over their labours without due attention and respect.

---

*An Introduction to the Study of Human Anatomy.* By James Paxton, Member of the Royal College of Surgeons, Honorary Member of the Ashmolean Society, Author of the Notes and Illustrations of Dr. Paley's Natural Theology, &c. Vol. I.; with numerous Illustrations.—8vo. pp. 415. Sherwood and Co., London; and Vincent, Oxford, 1831.

The principal object of this work is to furnish the student in anatomy with sufficient directions for cultivating this very important department of science in the shortest and most successful manner; and whoever examines it will, we confidently predict, agree with us, that Mr. Paxton has eminently succeeded in this intention. The text is accurate, concise, and very perspicuous; every part described is illustrated by a woodcut, *in the same page* with the descriptive text. By this judicious arrangement, the student will find his labours very much diminished and facilitated: he has immediately under his eye both graphic and descriptive anatomy, and is spared the wearisome task, which most other anatomical works inflict, of turning from the description to the plate, and from the plate to the description, until his mind is exhausted before he has gained the information he desires. Anatomical engravings, in general, also, contain such crowds of referential letters, figures, and marks, that the student is bewildered in his attempts to find them. In Mr. Paxton's work this fault is entirely avoided: but few parts are represented in each woodcut, and consequently but few letters of reference are required. The dullest capacity cannot fail to understand completely the brief, yet lucid, descriptions which are given of the structure and relative situation of the various parts; and the clearness and simplicity of the illustrations render them equally intelligible. This work may justly be said to make anatomy an easy and delightful study, and we shall be much mistaken if it does not speedily become the favorite anatomical guide of professional students, and of all those who are desirous of making themselves acquainted with the structure of the human body.

The opinion entertained of a work is often, perhaps, estimated by the length of the notice given of it: but it must be remembered, that many works, and "an Introduction to Anatomy" among the number, whatever may be their merit, will not admit of a detailed analysis or review. The first volume only is yet published, but we believe the work will speedily be completed.

---

